# Pioneering Klebsiella Pneumoniae Antibiotic Resistance Prediction With Artificial Intelligence-Clinical Decision Support System–Enhanced Matrix-Assisted Laser Desorption/Ionization Time-of-Flight Mass Spectrometry: Retrospective Study

**DOI:** 10.2196/58039

**Published:** 2024-11-07

**Authors:** Ming-Jr Jian, Tai-Han Lin, Hsing-Yi Chung, Chih-Kai Chang, Cherng-Lih Perng, Feng-Yee Chang, Hung-Sheng Shang

**Affiliations:** 1 Division of Clinical Pathology, Department of Pathology Tri-Service General Hospital National Defense Medical Center Taipei City Taiwan; 2 Graduate Institute of Medical Science National Defense Medical Center Taipei City Taiwan; 3 Division of Infectious Diseases and Tropical Medicine, Department of Medicine Tri-Service General Hospital National Defense Medical Center Taipei City Taiwan

**Keywords:** Klebsiella pneumoniae, multidrug resistance, AI-CDSS, quinolone, ciprofloxacin, levofloxacin

## Abstract

**Background:**

The rising prevalence and swift spread of multidrug-resistant gram-negative bacteria (MDR-GNB), especially *Klebsiella pneumoniae* (KP), present a critical global health threat highlighted by the World Health Organization, with mortality rates soaring approximately 50% with inappropriate antimicrobial treatment.

**Objective:**

This study aims to advance a novel strategy to develop an artificial intelligence-clinical decision support system (AI-CDSS) that combines machine learning (ML) with matrix-assisted laser desorption/ionization time-of-flight mass spectrometry (MALDI-TOF MS), aiming to significantly improve the accuracy and speed of diagnosing antibiotic resistance, directly addressing the grave health risks posed by the widespread dissemination of pan drug-resistant gram-negative bacteria across numerous countries.

**Methods:**

A comprehensive dataset comprising 165,299 bacterial specimens and 11,996 KP isolates was meticulously analyzed using MALDI-TOF MS technology. Advanced ML algorithms were harnessed to sculpt predictive models that ascertain resistance to quintessential antibiotics, particularly levofloxacin and ciprofloxacin, by using the amassed spectral data.

**Results:**

Our ML models revealed remarkable proficiency in forecasting antibiotic resistance, with the random forest classifier emerging as particularly effective in predicting resistance to both levofloxacin and ciprofloxacin, achieving the highest area under the curve of 0.95. Performance metrics across different models, including accuracy, sensitivity, specificity, positive predictive value, negative predictive value, and *F*_1_-score, were detailed, underlining the potential of these algorithms in aiding the development of precision treatment strategies.

**Conclusions:**

This investigation highlights the synergy between MALDI-TOF MS and ML as a beacon of hope against the escalating threat of antibiotic resistance. The advent of AI-CDSS heralds a new era in clinical diagnostics, promising a future in which rapid and accurate resistance prediction becomes a cornerstone in combating infectious diseases. Through this innovative approach, we answered the challenge posed by KP and other multidrug-resistant pathogens, marking a significant milestone in our journey toward global health security.

## Introduction

The World Health Organization (WHO) has highlighted antibiotic resistance as a grave global health threat, particularly emphasizing the challenge posed by multidrug-resistant *Klebsiella pneumoniae* (MDR-KP) [1]. Bacteremia caused by MDR-KP is linked to mortality rates as high as 47%, underlining the critical impact of multidrug resistance [2-4]. Initially, quinolones were highly effective against such infections due to their unique mechanism of action, distinct from β-lactams, targeting different bacterial processes [3,4]. However, increasing quinolone resistance among KP, driven by mutations in key enzyme regions, efflux pump overexpression, and plasmid-mediated mechanisms, undermines quinolone effectiveness, complicating infection management and underscoring the adaptability of bacterial resistance [3,4].

The emergence of quinolone resistance in KP strains within clinical settings indicates the critical demand for novel diagnostic techniques [2-7]. Rapid and accurate detection of antibiotic-resistant pathogens in patients is essential for controlling resistance spread [8,9]. Thus, the slow pace of culture-based diagnostics in addressing antibiotic resistance required shifting toward new, more agile strategies, emphasizing the need for quicker, more effective treatment options to navigate the challenges of rapidly evolving infections [10].

The dynamic battle against antibiotic resistance necessitates quick-to-adapt diagnostic tools for effective management [10]. Matrix-assisted laser desorption/ionization time-of-flight mass spectrometry (MALDI-TOF MS) is indispensable for the rapid and accurate identification of microorganisms [11,12]. Its application extends to the detection of virulence factors in antibiotic-resistant strains yet often transcends standard procedural bounds [11,12]. Combining machine learning (ML) with MALDI-TOF MS ushers in a novel paradigm for predicting antibiotic resistance [13], specifically targeting quinolone-resistant KP.

Our study introduces the development of an artificial intelligence-clinical decision support system (AI-CDSS) designed to enhance the clinical decision-making process regarding the use of quinolones as a potential antibiotic option. Integrating the precision of MALDI-TOF MS technology with advanced ML algorithms, our system aims to equip health care professionals with quick and evidence-based recommendations for the optimal use of antibiotics tailored to individual patient needs. This innovative approach not only improves the rapidity and precision of identifying antibiotic resistance but also offers clinicians valuable insights, enabling prompt and well-informed treatment choices. Consequently, this facilitates improved patient outcomes and more effective combat against the proliferation of antibiotic resistance.

## Methods

### Study Designs and Data Collection

From January 2021 to December 2023, a comprehensive research project was undertaken by the Tri-Service General Hospital alongside 4 district hospitals, examining 165,299 bacterial specimens using advanced MALDI-TOF MS technology provided by bioMérieux, France. This study identified 11,996 specimens as KP, which were further subjected to antibiotic sensitivity testing (AST) for levofloxacin (LEV) and ciprofloxacin (CIP) using the VITEK 2 system (bioMérieux). This system is claimed to be precise in such assessments. The analysis of the AST results strictly adhered to the clinical breakpoints defined by the Clinical and Laboratory Standards Institute, ensuring that the study aligned with the established microbiological norms [14,15].

The objective of this extensive analysis was to develop 2 predictive models: one to ascertain resistance to LEV and the other to CIP. The exclusion of 869 specimens from the LEV resistance analysis was necessary because of intermediate AST results, which refined the focus to 11,127 specimens for thorough investigation. Similarly, for CIP resistance, 373 specimens were excluded because of intermediate results, concentrating the analysis on 11,623 specimens. This detailed and systematic process emphasizes the rigor of the study and its commitment to deliver precise and reliable evaluations of antibiotic resistance among microbial specimens. The dataset was strategically divided as follows: samples collected from January to September were designated for training the models, whereas those collected from October to December served as the validation set. This temporal split was performed to simulate the application of the models in real-world settings.

### Data Preprocessing and Feature Extraction

Our research was initiated by extracting mass-to-charge (m/z) ratios and intensity values from MALDI-TOF MS data to establish an essential foundation. We addressed data imbalance through a resampling strategy using sklearn.utils, applying downsampling to over-represented classes and upsampling for underrepresented classes, thus balancing the dataset to diminish model bias and enhance generalization. In feature engineering, we segmented m/z ranges into 1-unit intervals, allowing a ± 2-unit tolerance for grouping similar observations. We then extracted and logarithmically transformed the peak intensity values for both the antibiotic-resistant and antibiotic-susceptible strains within these segments. This crucial step normalizes the data, improves strain comparability, and enables precise identification of resistance pattern variations. We filtered out segments with a feature importance value below 0.01 as background noise, focusing our model on significant attributes to optimize performance and achieve our research objectives.

### ML Model Development and Training

To detect LEV and CIP resistance in KP, we selected an array of sophisticated algorithms including Logistic Regression, Linear Discriminant Analysis, Random Forest, Gradient Boosting Classifier, AdaBoost Classifier, XGBoost, and light gradient boosting machine (LGBM). This diverse selection was aimed at leveraging their distinct strengths for a more precise and comprehensive analysis. Our goal was to thoroughly explore antibiotic resistance patterns to improve treatment strategies and provide valuable insights into the management of microbial resistance. A grid search was performed for each model to determine the optimal parameters, enhancing accuracy and performance. The best parameters identified for each model are detailed in Multimedia Appendix 1.

### Model Evaluation

To assess the accuracy of our predictive models, we used several key metrics, including receiver operating characteristic (ROC) curves, area under the curve (AUC), sensitivity, specificity, positive predictive value (PPV), negative predictive value (NPV), and *F*_1_-score. These metrics provide a detailed evaluation of the effectiveness of the model and ensure that the findings are reliable and robust. Such a comprehensive evaluation method is vital for predicting antibiotic resistance.

### Clinical Validation

To affirm the applicability of our models in real-world clinical settings, we conducted clinical validation at Tri-Service General Hospital using the validation dataset. During the validation period, we specifically validated 3339 cases of LEV resistance and 3487 cases of CIP resistance against traditional culture-based AST. We evaluated the predictive accuracy by analyzing sensitivity, specificity, PPV, NPV, and F1-score.

### AI-CDSS Deployment

**Figure 1:**
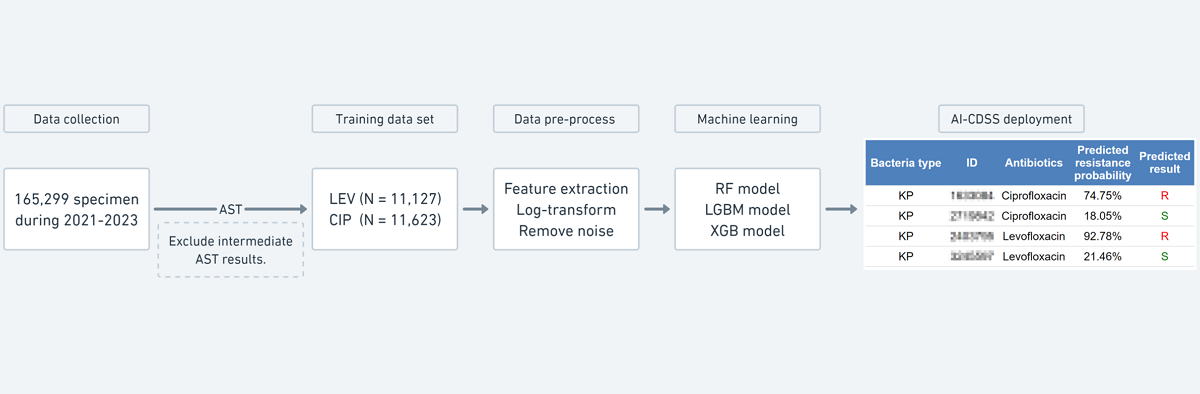
AI-CDSS development workflow. AI-CDSS: artificial intelligence-clinical decision support system; AST: antibiotic susceptibility testing; LEV: levofloxacin; CIP: ciprofloxacin; RF: random forest; LGBM: light gradient boosting machine; XGB: XGBoost.

We created a web-based AI-CDSS specifically designed for health care professionals. This system leverages cutting-edge ML algorithms to quickly determine the resistance to LEV and CIP. The process began with the collection of a culture sample, which the AI-CDSS then analyzed to predict resistance patterns. For a clear understanding of our process, The flowchart in Figure 1 represents the systematic process behind the development of the AI-CDSS, which commenced with a comprehensive data collection phase, acquiring 165,299 bacterial specimens from January 2021 to December 2023. Post identification of *Klebsiella pneumoniae* within the samples, they were subjected to AST. Specimens yielding intermediate AST results were excluded, resulting in 11,127 for LEV and 11,623 for CIP resistance analyses, thus forming a training dataset. Subsequent data preprocessing involved meticulous feature extraction and log transformation of mass spectrometry data to eliminate noise and standardize the dataset. This dataset was then used to train robust ML models, such as Random Forest, LGBM, and XGBoost, to accurately predict antibiotic resistance. The AI-CDSS, by leveraging these models, facilitates rapid and precise predictions of LEV and CIP resistance, ultimately aiding health care professionals in informed clinical decision-making.

### Ethical Considerations

This retrospective study adhered to the ethical standards outlined in the Declaration of Helsinki and relevant local regulations for human subject protection. The institutional review board of Tri-Service General Hospital, Taipei, Taiwan, approved the research (TSGHIRB No. C202305073). Due to the study's retrospective design, the requirement for informed consent was waived. All analyzed data were anonymized to ensure patient confidentiality.

## Results

### ML Model Development Using MS Data

**Figure 2:**
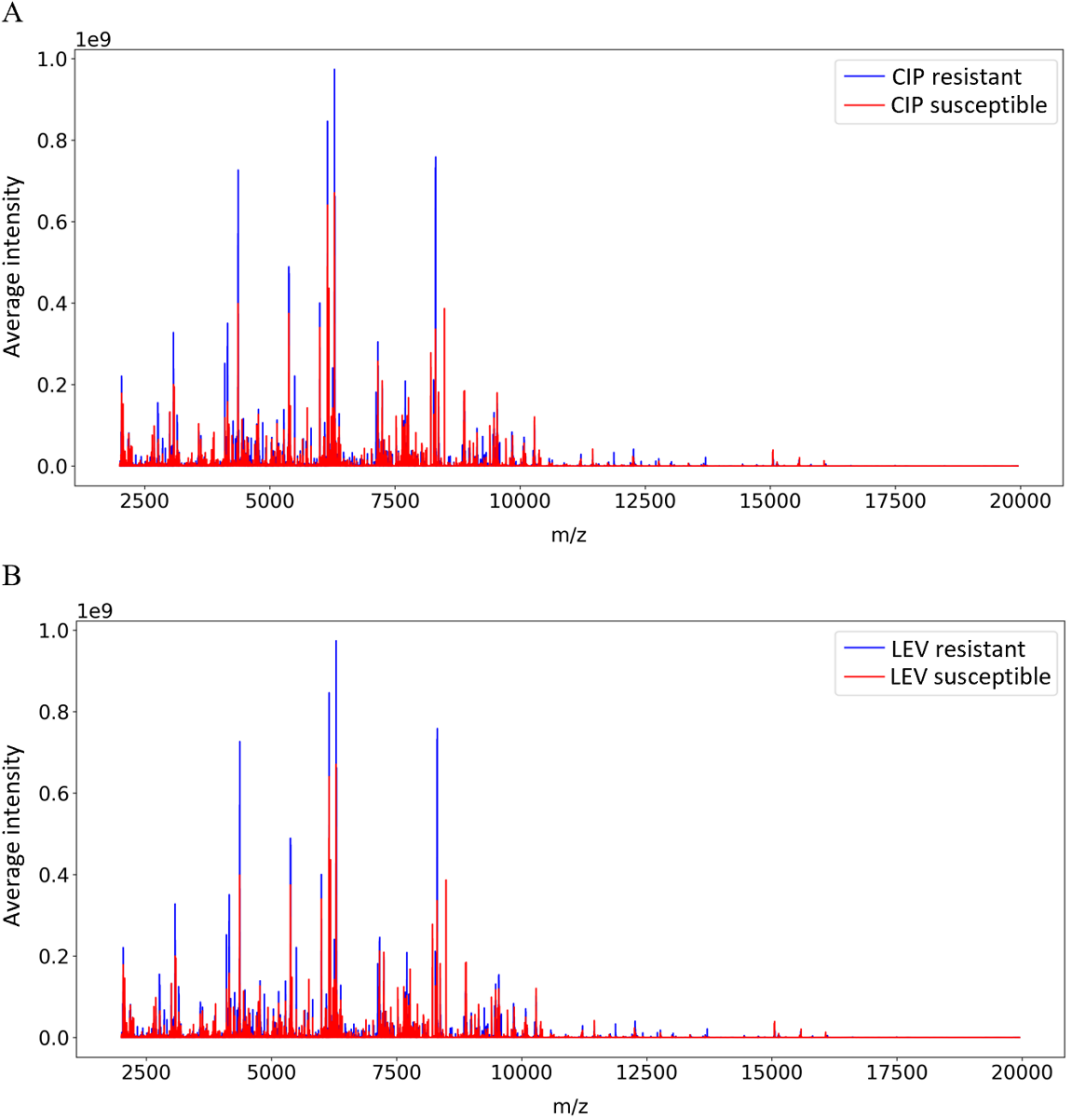
Differential mass spectrometry profiles for CIP and LEV resistance in Klebsiella pneumoniae. (A) Average intensity distribution for CIP-resistant and susceptible Klebsiella pneumoniae. (B) Average intensity distribution for LEV-resistant and susceptible Klebsiella pneumoniae. CIP: ciprofloxacin; LEV: levofloxacin; m/z: mass-to-charge.

MS analyses revealed distinct spectral differences that differentiated antibiotic-resistant KP strains from their susceptible counterparts. Figure 2 shows the average intensity distributions across m/z segments for KP strains, with CIP resistance shown in blue and susceptibility in red. The top graph (A) highlights the m/z segments that differ between CIP-resistant and susceptible strains, whereas the bottom graph (B) depicts analogous distributions for LEV resistance. These graphs provide a stark visual comparison of the spectral differences, emphasizing the distinct spectral markers associated with antibiotic resistance that can be instrumental for rapid resistance detection.

MS data for KP showed a consistent array of the top 15 spectral features in strains resistant to both CIP and LEV. These features span m/z ratio ranges of 2035-2040, 2067-2072, 2166-2171, 2180-2185, 2688-2695, 2759-2764, 3143-3152, 4363-4368, 4567-4572, 5278-5283, 5379-5384, 7238-7253, 7703-7708, 9136-9145, and 9532-9544. The shared features across CIP- and LEV-resistant strains suggest a universal mechanism of resistance despite the different modes of action of antibiotics.

**Figure 3:**
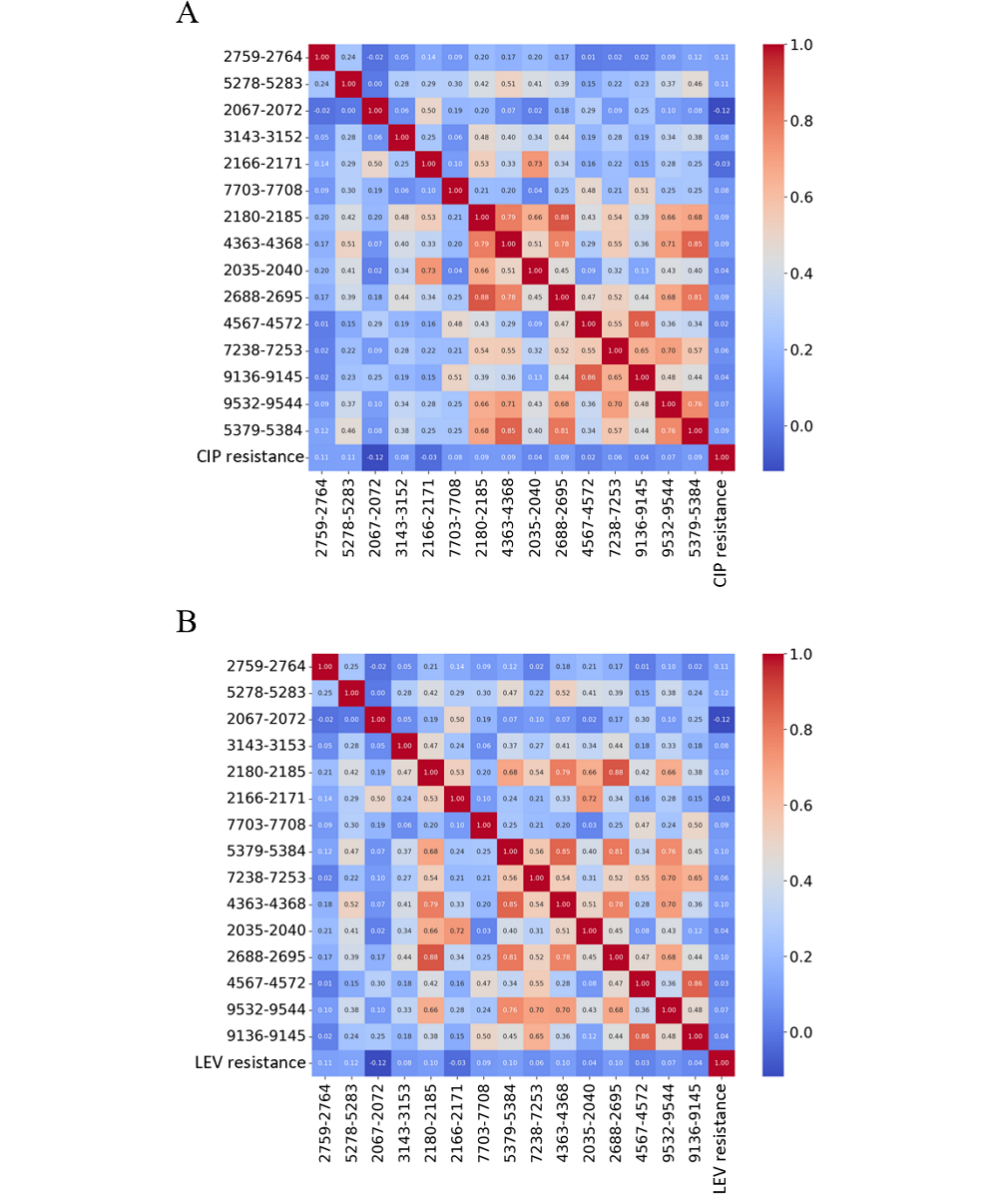
Correlation heatmaps of Top 15 m/z features for CIP and LEV resistance in Klebsiella pneumoniae. (A) Correlation heatmap for CIP resistance. (B) Correlation heatmap for LEV resistance. m/z: mass-to-charge; CIP: ciprofloxacin; LEV: levofloxacin.

This is graphically represented in the heat maps in Figure 3A for ciprofloxacin and Figure 3B for levofloxacin, where the correlation between each m/z ratio range and resistance status are color-coded for clarity. The intensity of the color correlates with the strength of the association; the redder the hue, the stronger the correlation with resistance. These heat maps provide a visual summary of the data, emphasizing the features most strongly associated with resistance, thus informing future molecular studies and aiding in the development of new approaches to combat antibiotic resistance. In Figure 3, The top graph (A) shows the correlation between the top 15 m/z segments and CIP resistance, whereas the bottom graph (B) illustrates these correlations for LEV resistance. The heatmaps transition from blue to red, indicating an increasing strength of correlation from weaker to stronger, respectively. These patterns highlighted the top 15 differential m/z segments that were most indicative of antibiotic resistance.

### Model Performance Metrics, Validation Results, and ROC Curve Analyses

To assess the efficacy of ML models in predicting antibiotic resistance in KP, we evaluated several algorithms across 2 different drug resistances. The model performance metrics, including the AUC, accuracy, sensitivity, specificity, PPV, NPV, and *F*_1_-score, are detailed in Tables 1 and 2 for CIP and LEV resistance, respectively. For CIP resistance, the Random Forest demonstrated the highest testing AUC at 0.95, indicative of its robust predictive power, with Gradient Boosting Classifier and XGBoost also performing notably well with AUCs of 0.95 and 0.94, respectively. These models are particularly useful in identifying CIP-resistant KP strains with high accuracy and precision.

**Table 1:** Performance metrics of machine learning models for ciprofloxacin resistance.

Models	Training AUC^a^	Testing AUC	Accuracy	Sensitivity	Specificity	PPV^b^	NPV^c^	*F*_1_-score
RF^d^	0.99	0.95	0.87	0.88	0.86	0.81	0.91	0.85
LGBM^e^	0.99	0.95	0.90	0.88	0.91	0.87	0.92	0.88
GBC^f^	0.99	0.95	0.87	0.92	0.84	0.80	0.93	0.86
XGBoost	0.99	0.95	0.87	0.89	0.86	0.82	0.92	0.85
AdaBoost	0.99	0.87	0.77	0.81	0.74	0.69	0.85	0.74
LR^g^	0.91	0.84	0.78	0.77	0.78	0.71	0.83	0.74
LDA^h^	0.96	0.80	0.77	0.80	0.75	0.69	0.84	0.74

^a^AUC: area under the curve.

^b^PPV: positive predictive value.

^c^NPV: negative predictive value.

^d^RF: random forest.

^e^LGBM: light gradient boosting machine.

^f^GBC: gradient boosting classifier.

^g^LR: logistic regression.

^h^LDA: linear discriminant analysis.

**Table 2:** Performance metrics of machine learning models for Levofloxacin resistance.

Models	Training AUC^a^	Testing AUC	Accuracy	Sensitivity	Specificity	PPV^b^	NPV^c^	*F*_1_-score
RF^d^	0.99	0.95	0.86	0.89	0.82	0.86	0.86	0.87
GBC^e^	0.99	0.95	0.87	0.88	0.86	0.89	0.85	0.88
XGBoost	0.99	0.93	0.85	0.86	0.85	0.87	0.83	0.86
LGBM^f^	0.99	0.92	0.85	0.84	0.85	0.87	0.82	0.86
AdaBoost	0.99	0.90	0.85	0.85	0.84	0.87	0.83	0.86
LR^g^	0.77	0.73	0.66	0.74	0.57	0.68	0.64	0.70
LDA^h^	0.72	0.68	0.64	0.81	0.43	0.63	0.65	0.71

^a^AUC: area under the curve.

^b^PPV: positive predictive value.

^c^NPV: negative predictive value.

^d^RF: random forest.

^e^GBC: gradient boosting classifier.

^f^LGBM: light gradient boosting machine.

^g^LR: logistic regression.

^h^LDA: linear discriminant analysis.

**Figure 4:**
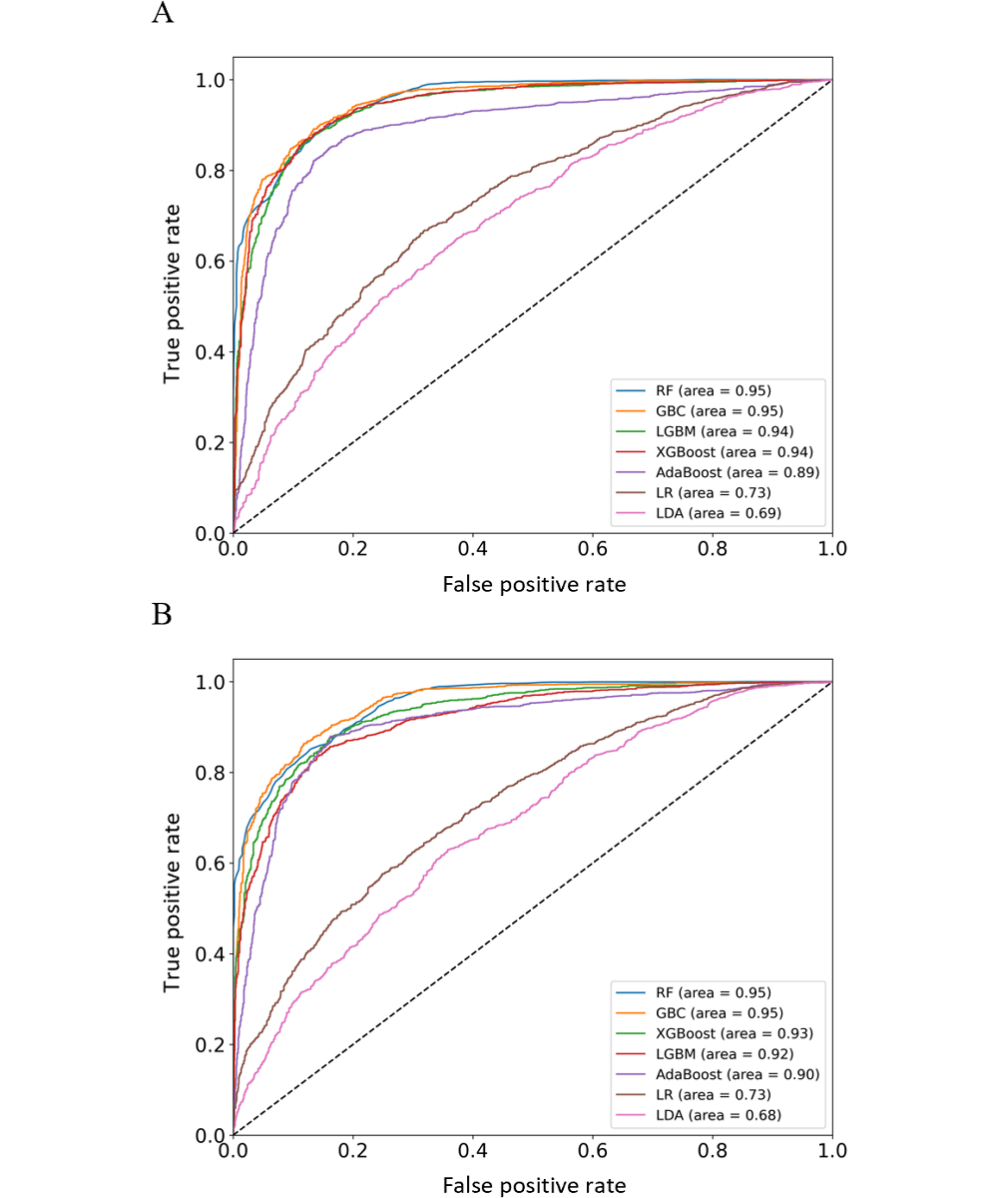
ROC curves for predicting antibiotic resistance in Klebsiella pneumoniae. (A) ROC curves for CIP resistance prediction. (B) ROC curves for LEV resistance prediction. ROC: receiver operating characteristic; CIP: ciprofloxacin; LEV: levofloxacin; RF: random forest; GBC: gradient boosting classifier; LGBM: light gradient boosting machine; LR: logistic regression; LDA: linear discriminant analysis.

The LEV resistance models displayed a similar trend, with the Random Forest classifier again showing exceptional performance, with an AUC of 0.95. The gradient boosting classifier and XGBoost showed similar effectiveness, with AUCs of 0.95 and 0.93. These algorithms proved effective in distinguishing LEV-resistant and LEV-susceptible strains with considerable accuracy. The ROC curves, as presented in Figures 4A and B, visually encapsulate the model performance, with the Random Forest, XGBoost, and Gradient Boosting classifiers exhibiting steep ascents toward the upper left corner, denoting high true-positive rates and low false-positive rates. Although logistic regression and Linear Discriminant Analysis showed lower AUC values, their inclusion offered a broad perspective and contributed to the ensemble approach in model evaluation. In Figure 4, the 2 charts display the performance of various ML models in classifying *Klebsiella pneumoniae* strains as resistant or susceptible to 2 antibiotics: CIP and LEV. The top panel (A) displays the ROC curves for models predicting CIP resistance, with the Random Forest classifier achieving an AUC of 0.95, indicating its excellent predictive ability. The bottom panel (B) displays the ROC curves for models predicting LEV resistance, where the random forest (RF) classifier also achieves a high AUC of 0.95, demonstrating consistently high performance across different antibiotic resistances. The curves plot each model's true positive rate (sensitivity) against its false positive rate (1-specificity), with the diagonal line representing a random chance. The closer a curve follows the left-hand border and then the top border of the ROC space, the more accurate the test.

Despite their range of performance metrics, our ensemble of models collectively provided a robust method for assessing KP resistance to CIP and LEV. This comprehensive approach, which leverages the strengths of multiple predictive algorithms, offers valuable insights into antibiotic resistance patterns, aiding the development of precision treatment strategies using our AI-CDSS.

### Integration of AI-CDSS in the Clinical Process

The integration of our AI-CDSS platform into the antibiotic resistance–testing workflow represents a significant advancement in the treatment of infectious diseases. This system streamlines the evaluation process from the initial culture of samples to the final decision-making in clinical settings. Using advanced ML algorithms, the platform rapidly predicts resistance to antibiotics, such as LEV and CIP, providing clinicians with precise and efficient analyses that inform treatment strategies. This is particularly critical for managing cases of KP, for which timely intervention is crucial. The platform’s predictive capabilities allow for resistance determination for approximately 3 days, a notable improvement over the conventional 4-day timeline required for standard AST. By accelerating the decision-making process, the AI-CDSS contributes to improved patient outcomes and enhances antibiotic stewardship efforts, ensuring that treatments are both appropriate and effective. The workflow diagram clearly contrasts the AI-CDSS with traditional AST methods, highlighting the reduction in time to clinical decisions and underscoring the benefits of real-time data in the management of infectious diseases.

## Discussion

### Principal Findings

This investigation of the predictive capabilities of ML and MALDI-TOF MS for identifying quinolone-susceptible KP and quinolone-resistant KP signifies a groundbreaking development in the expedited diagnosis of infectious diseases. The synergy between MALDI-TOF MS and ML provides an innovative approach to detecting and managing antibiotic-resistant pathogens. This advancement is particularly relevant for low- and middle-income countries (LMICs), where the WHO has noted a rapid escalation in antibiotic use, presenting challenges to access and stewardship [1,16]. Our findings are of significant value in LMICs, as they propose a strategic balance between antibiotic availability and stewardship, which could influence policy adaptations aimed at fulfilling WHO objectives and strengthening global health security against antimicrobial resistance.

The increasing prevalence of antibiotic resistance, notably in quinolone-resistant KP strains, necessitates novel diagnostic methods [2-9]. Traditional methods fail to swiftly identify these bacteria, thereby prolonging the initiation of suitable treatments and containment actions [10]. Previous research on ML with MALDI-TOF for discerning quinolone-resistant KP has used a modest number of isolates. Our study compiles an unprecedentedly large dataset for a ML model to detect quinolone-resistant KP, demonstrating the potential of advanced technologies in predicting resistance with high precision, thereby expediting accurate diagnoses and enabling effective treatments. The incorporation of algorithms such as Random Forest and XGBoost has shown high efficacy in differentiating between resistant and susceptible KP strains, playing a pivotal role in informed antibiotic selection and combating the spread of resistance.

### Comparative Advantages of MALDI-TOF MS and Next-Generation Sequencing in Clinical Settings

In clinical practice, KP was identified using MALDI-TOF MS and subsequently tested for antibiotic susceptibility with the VITEK 2 system. In our study, we used artificial intelligence to analyze MALDI-TOF MS spectra and predict KP as resistant or susceptible phenotypes. Although next-generation sequencing (NGS) is commonly used for genotype analysis in other studies, genotypic data may not always align with phenotypic expressions. Antibiotic resistance typically develops through genetic alterations, either by the acquisition of resistance genes or mutations in elements crucial for antibiotic activity. However, resistance can also occur without any genetic changes, a phenomenon known as phenotypic resistance illustrating the challenges of predicting antibiotic resistance based solely on genetic information [17-19]. Discrepancies may arise from pseudogenes in low antibiotic environments, where nonlethal mutations persist in contrast to the rapid elimination of ineffective genes in high antibiotic environments [18]. In addition, gene interactions, regulatory mechanisms, and expression levels significantly impact resistance gene functionality [19-21]. Our study emphasizes the ability of MALDI-TOF MS to quickly provide crucial, actionable resistance profiles for urgent clinical decisions [22,23]. NGS can provide a detailed analysis of resistance genes; however, its longer processing times limit its use in emergencies [19-21]. In contrast, MALDI-TOF MS can detect these mechanisms through its protein expression analysis, proving invaluable in clinical settings that require rapid decision-making [22,23].

### Speeding Up Pathogen Diagnostics With AI and MALDI-TOF MS

Our integration of AI-CDSS with MALDI-TOF MS technology offers a significant improvement in diagnostic speed and efficiency that answers the need for rapid diagnostics for infectious diseases, particularly those involving drug-resistant pathogens such as KP. Our approach reduces the time needed to determine antibiotic resistance to approximately 72 hours from the initial culture. Traditional culturing methods, while reliable, require up to 96 hours—72 hours for culturing and identification and an additional 24 hours for AST interpretation, potentially delaying critical treatment decisions [10,24]. In comparison, polymerase chain reaction (PCR) and sequencing methods still require 72 hours for the initial pathogen identification by MALDI-TOF MS, followed by 1-2 days for PCR and 3-4 days for sequencing, depending on the complexity of the analysis [20,21,25,26]. Comparisons of the timelines for each method were provided in Multimedia Appendix 2. Therefore, our study demonstrates that the integration of AI-CDSS with MALDI-TOF MS not only significantly speeds up the diagnostic process but also improves the timeliness of clinical responses, crucial for managing severe infections.

### Impact and Future Directions

In addition to the advancements detailed above, the integration of an AI-CDSS is a groundbreaking enhancement of our diagnostic capabilities. The AI-CDSS, which leverages the predictive power of ML models, offers clinicians real-time, data-driven insights, significantly improving the decision-making process in the treatment of infections caused by LEV- and CIP-resistant KP. This system can rapidly interpret complex diagnostic data, recommend personalized treatment options, and predict potential resistance patterns, thereby streamlining the diagnostic workflow and facilitating a more targeted approach to antibiotic therapy. The introduction of AI-CDSS into our methodology underscores our commitment to harness cutting-edge technology to combat antibiotic resistance, representing a vital step forward in the optimization of clinical outcomes and advancement of antibiotic stewardship.

### Limitations

This study underscores the intricacies involved in dataset balancing and the pivotal role of feature selection within the domain of ML algorithms. A comprehensive approach to data preprocessing, feature extraction, and the subsequent training of models delineates the multifaceted nature of constructing dependable predictive frameworks. In addition, intermediate results often present challenges in predictive models due to the uncertainty they introduce, which can potentially skew the model’s outcomes. Consequently, the exclusion of specimens with intermediate AST results (869 for LEV and 373 for CIP) allowed for a more focused study but potentially omitted significant data, affecting model generalizability for these intermediate cases. Future research should focus on enhancing these models by incorporating intermediate cases, as well as diverse data modalities, to broaden their applicability and encompass a wider array of antibiotic-resistant organisms. Furthermore, the significance of this investigation transcends the boundaries of immediate clinical use and serves as a cornerstone for informing public health policies and shaping infection control methodologies. This study makes a substantial contribution to international efforts aimed at combating antimicrobial resistance by providing a swift, precise, and economically viable mechanism to identify antibiotic resistance. This issue remains a global public health priority and requires continued attention and action.

### Comparison With Previous Work

Previous studies used conventional PCR-based methods to identify quinolone resistance genes among KP clinical isolates [27,28]. ML techniques combined with MALDI-TOF of discrimination of carbapenem-resistant KP with limited sample isolates [29,30]. To date, there are no studies discussing the use of ML combined with MALDI-TOF for the rapid identification of quinolone resistance in KP. Our research establishes the most extensive collection of isolates to date for constructing a ML model aimed at identifying LEV and CIP resistance. The methodological innovation of this study, through the analysis of approximately 12,000 KP cultures, demonstrates the feasibility and accuracy of using advanced technologies to predict resistance patterns. This approach not only enhances the precision of diagnostics but also significantly reduces the time required to identify resistant strains, facilitating timely and targeted therapeutic interventions.

### Conclusions

MALDI-TOF MS is a swift, accurate, and cost-efficient method for identifying bacteria, including its application in the detection of antibiotic resistance. This study highlights the successful AI-CDSS with the application of a ML-enhanced MALDI-TOF approach for predicting quinolone-resistant KP. However, certain constraints exist, particularly when resistance mechanisms are not mediated at the protein-peptide level or fall outside the m/z ratio range of 2000-20,000. Future studies are required to validate the results further.

## Appendix

Multimedia Appendix 1 Parameters of machine learning models for predicting Levofloxacin and Ciprofloxacin resistance.

Multimedia Appendix 2 Workflow comparison of diagnostic techniques for antibiotic resistance detection.

## Abbreviations

AI-CDSS: artificial intelligence-clinical decision support system
AST: antibiotic sensitivity testing
AUC: area under the receiver operating characteristic curve
CIP: ciprofloxacin
KP: Klebsiella pneumoniae
LEV: levofloxacin
LGBM: light gradient boosting machine
LMIC: low- and middle-income country
m/z: mass-to-charge ratio
MALDI-TOF MS: matrix-assisted laser desorption/ionization time-of-flight mass spectrometry
MDR-GNB: multidrug-resistant gram-negative bacteria
MDR-KP: multidrug-resistant Klebsiella pneumoniae
ML: machine learning
NGS: next-generation sequencing
NPV: negative predictive value
PCR: polymerase chain reaction
PPV: positive predictive value
ROC: receiver operating characteristic
WHO: World Health Organization

## Data Availability

The datasets generated during and/or analyzed during this study are available from the corresponding author on reasonable request.
