# Supplementary material for: Pioneering Klebsiella Pneumoniae Antibiotic Resistance Prediction With Artificial Intelligence-Clinical Decision Support System–Enhanced Matrix-Assisted Laser Desorption/Ionization Time-of-Flight Mass Spectrometry: Retrospective Study
Source: J Med Internet Res. 2024 Nov 7;26:e58039. doi: 10.2196/58039 (PMC11582491; doi:10.2196/58039)
Supplement: Multimedia Appendix 1 [file jmir_v26i1e58039_app1.docx]

**Multimedia Appendix 1. Parameters of Machine Learning Models for Predicting Levofloxacin and Ciprofloxacin Resistance**

| **Model** | **Parameter** | **LEV Models** | **CIP Models** |
| --- | --- | --- | --- |
| **RF** | n_estimators | 300 | 200 |
|  | max_depth | 30 | 10 |
|  | min_samples_split | 10 | 2 |
|  | min_samples_leaf | 1 | 2 |
|  | max_features | sqrt | sqrt |
|  | bootstrap | False | True |
| **GBC** | n_estimators | 300 | 300 |
|  | max_depth | 7 | 7 |
|  | min_samples_split | 2 | 6 |
|  | learning_rate | 0.1 | 0.1 |
|  | subsample | 0.8 | 0.6 |
| **XGBoost** | n_estimators | 300 | 300 |
|  | max_depth | 7 | 7 |
|  | learning_rate | 0.05 | 0.1 |
|  | colsample_bytree | 0.6 | 0.6 |
|  | subsample | 0.8 | 1.0 |
| **LGBM** | n_estimators | 200 | 300 |
|  | max_depth | 15 | 10 |
|  | learning_rate | 0.1 | 0.1 |
|  | num_leaves | 70 | 31 |
| **AdaBoost** | n_estimators | 200 | 200 |
|  | max_depth | 3 | 3 |
|  | learning_rate | 1 | 1 |
|  | base_estimator | DecisionTreeClassifier | DecisionTreeClassifier |

Abbreviations: CIP, Ciprofloxacin; GBC, Gradient Boosting Classifier; LEV, Levofloxacin; LGBM, Light Gradient Boosting Machine; RF, Random Forest
