# Supplementary figures and images for: Pioneering Klebsiella Pneumoniae Antibiotic Resistance Prediction With Artificial Intelligence-Clinical Decision Support System–Enhanced Matrix-Assisted Laser Desorption/Ionization Time-of-Flight Mass Spectrometry: Retrospective Study
Source: J Med Internet Res. 2024 Nov 7;26:e58039. doi: 10.2196/58039 (PMC11582491; doi:10.2196/58039)

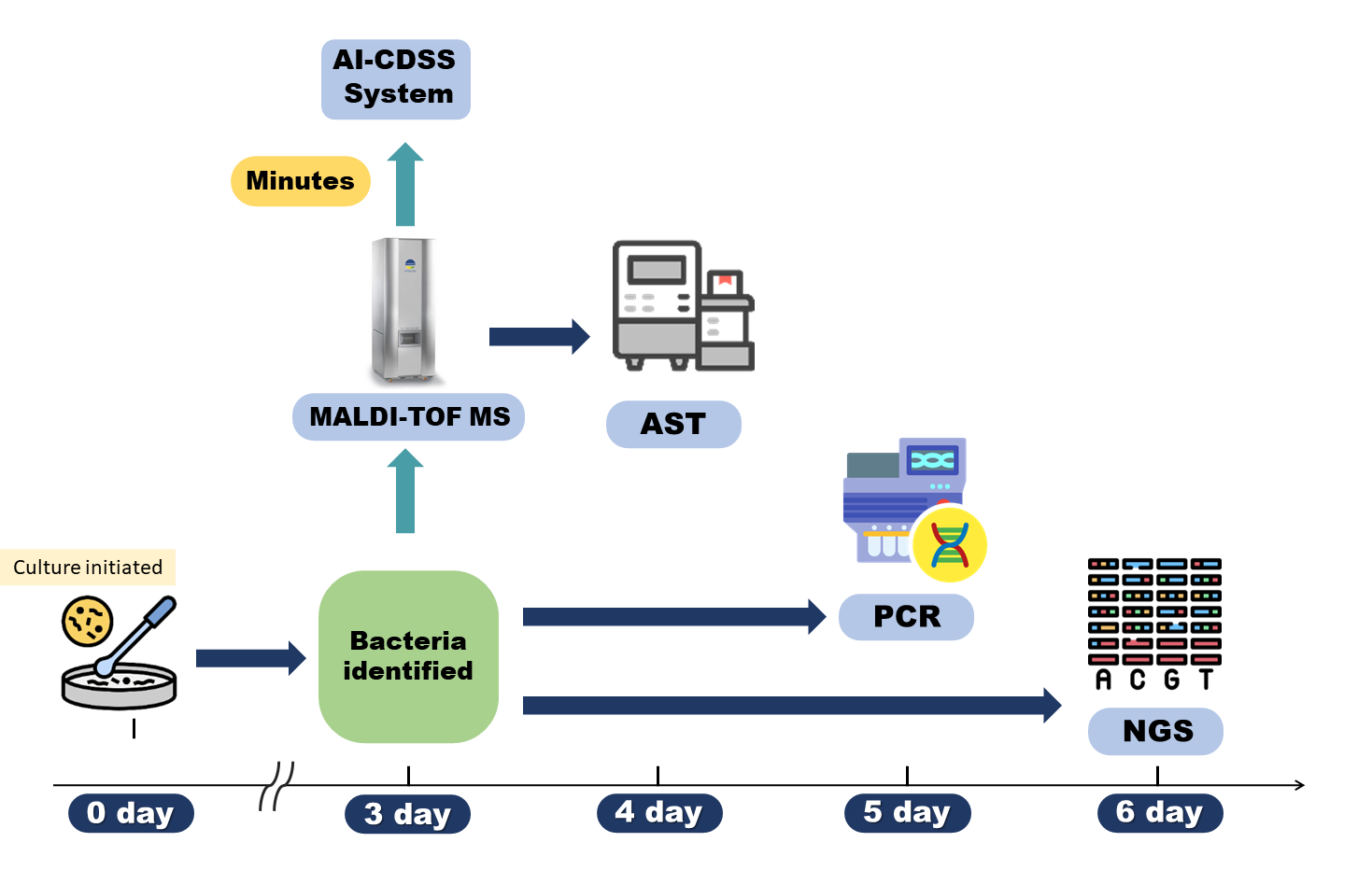

Supplement: Multimedia Appendix 2 [file jmir_v26i1e58039_app2.png]
